# Hepatitis C core antigen testing to diagnose active hepatitis C infection among haemodialysis patients

**DOI:** 10.1186/s12882-020-02154-4

**Published:** 2020-11-13

**Authors:** Xue Zheng Wong, Chye Chung Gan, Rosmawati Mohamed, Rosnawati Yahya, Shubash Ganapathy, Soek Siam Tan, Soo Kun Lim

**Affiliations:** 1 Department of Medicine, Faculty of Medicine, University of Malaya, Jalan University, 50603 Kuala Lumpur, Malaysia; 2 Hospital Kuala Lumpur, Jalan Pahang, 50586 Kuala Lumpur, Malaysia; 3 National Institutes of Health, Ministry of Health, Kompleks Institut Kesihatan Negara (NIH), No. 1, Jalan Setia Murni U13/52 Seksyen U13, Setia Alam, 40170 Shah Alam, Selangor Malaysia; 4 Selayang Hospital, B21, Lebuhraya Selayang - Kepong, 68100 Batu Caves, Selangor Malaysia; 5 8TE, Menara Timur, Pusat Perubatan Universiti Malaya, Jalan Universiti, Lembah Pantai, 50603 Kuala Lumpur, Malaysia

**Keywords:** Abbott ARCHITECT HCV Ag, HCV Core antigen, Hepatitis C virus, HCV RNA, Haemodialysis

## Abstract

**Background:**

Hepatitis C virus (HCV) infects more than 71 million people worldwide and chronic HCV infection increases the risk of liver cirrhosis and failure. Haemodialysis (HD) is one of the renal replacement therapies with risk of HCV transmission. Anti-HCV antibodies are the serological screening test for HCV infection that does not detect active phase of infection. Majority HCV infected HD patients in Malaysia do not have further HCV RNA performed due to high cost and thus HCV treatment is less frequently offered. HCV Core Antigen (HCV Ag) can potentially be used to diagnose active HCV infection in HD population in comparison to HCV RNA, at lower cost.

**Methods:**

We conducted a cross-sectional study to assess the correlation between HCV Ag and HCV RNA and to identify the prevalence of active HCV infection among HCV seropositive HD patients from dialysis centres across West Malaysia from July 2019 to May 2020. Pre-dialysis blood was taken and tested for both HCV Ag and HCV RNA tests. HCV Ag was tested with Abbott ARCHITECT HCV Ag test.

**Results:**

We recruited 112 seropositive HD patients from 17 centres with mean age of 54.04 ± 11.62 years, HD vintage of 14.1 ± 9.7 years, and male constitute 59.8% (67) of the study population. HCV Ag correlates well with HCV RNA (Spearman test coefficient 0.833, *p* < 0.001). The sensitivity was 90.7%, specificity 100%, positive predictive value (PPV) 100%, negative predictive value (NPV) 76.5%, and accuracy 92.9%. For HCV RNA level > 3000 IU/mL, HCV Ag had a higher sensitivity of 95.1% and greater correlation (Spearman test coefficient 0.897, p < 0.001). The prevalence of active HCV infection was 76.8% among HCV seropositive HD patients.

**Conclusions:**

Although HCV Ag is less sensitive, it shows an excellent correlation with HCV RNA and has 100% PPV. HCV Ag can be considered as an alternative diagnostic tool for chronic active HCV infection among HD cohort, who can then be considered for HCV treatment. For seropositive HD patient with negative HCV Ag, we recommend to follow-up with HCV RNA test.

**Supplementary Information:**

The online version contains supplementary material available at 10.1186/s12882-020-02154-4.

## Background

Hepatitis C virus (HCV) causes a significant worldwide burden and infects more than 185 million people worldwide, in which 71 millions of them are chronically infected [1]. HCV infection has caused a major burden from its sequelae of chronic infection. With current direct-acting antivirals (DAA) that accomplish high sustained virological response (> 95%), WHO had pursued a goal to eradicate HCV as a public health threat by 2030 [2]. Despite having good treatment options, most of the people with HCV are undiagnosed, which leads to a low treatment rate. This is specifically observed in low- and middle-income countries (LMICs), such as Malaysia with limited resources and many competing health needs from its citizens to organize a full-scale, nationwide initiatives to eliminate HCV. Hence, a concept of “Micro-elimination”, which entails a phased approach to achieve national elimination goals in defined segments of the population through the engagement of multi-stakeholders who are most knowledgeable about the needs of the specific populations to tailor interventions within these settings to eliminate HCV [3].

Chronic haemodialysis (HD) patients are a population that should be prioritized as they have higher HCV prevalence rate, higher risk of transmitting HCV to others, and readily identified by mandatory screening. Both rigorous screening and early treatment of HCV in HD patients are crucial in reducing the incidence and outbreak in this cohort, in line with the paradigm of “treatment as prevention” with the ultimate goal of micro-elimination of HCV among this cohort [3].

Anti-HCV antibody is the serological screening test for HCV infection which is recommended by WHO [2]. Although it is relatively simple and cheap, it creates a great challenge to detect patients in acute phase of infection due to long “window period” (range: 45–68 days for cohort of non-HD patients [4], and median of 154 days for cohort of HD patients [5]) on top of the possibility of false negative anti-HCV in immunocompromised individuals inclusive of end-stage kidney disease. A confirmatory test is required to distinguish spontaneously resolved cases from chronic infection or active HCV infections [6]. Serum HCV RNA is widely used as a confirmatory test, but HCV RNA test is expensive and requires certain amount of technical skill to avoid false positive results as well as longer turnaround time [6, 7]. Hence, a significant number of anti-HCV positive patients failed to proceed with confirmatory HCV tests and are often lost to follow-up, leading to a low treatment rate, especially among HD populations [8].

In contrast, HCV Core Antigen (HCV Ag) has been shown to be a dependable marker to diagnose active infection [9], with lower cost and shorter processing time [6, 10]. Additionally, it is used to assess treatment response to direct antiviral agents [9, 11]. Furthermore, in the era of new interferon-free, oral direct antiviral therapy that consists of pan-genotypic regime, HCV Ag alone is sufficient for diagnosis of active HCV infection, despite its lower analytical sensitivity as compared to HCV RNA [7]. Up to date, using HCV RNA test as confirmatory test for seropositive HD patients is still the main practice worldwide [12, 13]. Most studies using HCV Ag as a confirmatory test mainly involved general population. Although there are few studies on HCV Ag in HD cohort that show good correlation [6, 14, 15], these studies are limited and do not include Asians population, more specifically Malaysian population with multi-ethnicity. In Asia, HCV Ag availability is limited, where Malaysia is among the countries considering HCV Ag as an alternative to nucleic acid test. Whereas HCV Ag is still unavailable in Hong Kong or Singapore, and is relatively expensive in mainland China [13].

Recent studies have revealed that a combination of anti-HCV screening along with sequential confirmation with HCV Ag then HCV RNA on antigen negative could offer equivalent or greater diagnostic capability at a better cost. It is also a cost-effective screening algorithm to detect active HCV infections in high prevalence settings [16–18]. Locally, it is estimated that HCV Ag test is approximately 3 times cheaper than HCV RNA.

Hence, this study is designed to assess the correlation of HCV Ag with HCV RNA among HD cohort in our multi-ethnic population. Besides, we would like to identify the prevalence of active HCV viremia among HCV serology positive HD patients. This has important clinical implications for the planning of resource allocation to treat active HCV infection.

## Methods

### Study design

We conducted a cross-sectional study from July 2019 to May 2020 from 17 dialysis centres across West Malaysia. One hundred and twelve HCV seropositive patients were recruited based on their HCV serology result reported on Malaysia Dialysis and Transplantation Registry (MDTR). The inclusion criteria was End Stage Kidney Disease (ESKD) patients undergoing regular haemodialysis treatment. Refusal to participate or patients who are receiving treatment or have been treated for hepatitis C were excluded.

Patients’ anti-HCV antibody status was obtained from dialysis centres’ regular blood screening results. Patients’ data, which include demographics, medical and dialysis history, laboratory tests, and concomitant medications, were collected in a standardized data collection form.

### Blood sample collection

For all consented patients, blood was taken prior to their dialysis. Both HCV Ag and HCV RNA were being tested. Samples with HCV Ag 3 to 10 fmol/L would have a repeat testing using the same sample to confirm the results (Additional file 1).

### HCV Ag measurement

The HCV Ag was tested with the Abbott ARCHITECT HCV Ag test. This is a chemiluminescent microparticle immunoassay (CMIA). It uses microparticles coated with monoclonal anti-HCV to detect the HCV core Ag (nucleocapsid pepside 22, p22). HCV Ag level of ≥3.00 fmol/L was recognised as reactive whereas level < 3 fmol/L was recognised as non-reactive. This cut-off level of 3fmol/l has been shown to have high sensitivity and specificity as well as good correlation with HCV RNA > 3000 IU/ml.

### HCV RNA measurement

HCV RNA quantification was quantified with the Reverse Transcription Polymerase Chain Reaction (RT-PCR) on Qiagen Artus HCV QS-RGQ kit. The lower limit of detection was 21 IU/mL. Samples with HCV RNA level ≥ 21 IU/mL and < 21 IU/mL were reported as positive and negative, respectively. RT-PCR was considered as the gold standard method to compare the result of HCV Ag.

### Statistical analysis

The data was analyzed, whereby the descriptive analysis was done to report the continuous variables in mean and standard deviation, and categorical variables were reported in frequency and percentage. The sensitivity, specificity, positive predictive value, and negative predictive value were calculated to determine the accuracy of HCV Ag test compared to HCV RNA as gold standard. The correlation coefficients between HCV Ag and HCV RNA were calculated using Spearman’s rank test. Statistical significance was set at *p* value less than 0.05. Data were analyzed using SPSS for Macintosh Version 24.0 (SPSS, Inc., Chicago, IL, USA).

## Results

A total of 112 patients were successfully recruited into this study. Most of the patients were males (59.8%) with the mean age of 54.04 ± 11.62 years. Table 1 showed the demographic characteristics of the patients together with their primary cause of ESKD.

**Table 1:** Demographic and clinical characteristics of 112 patients recruited

Patient Characteristics	Frequency (%) / Mean ± SD
Gender	
Males	67 (59.8)
Females	45 (40.2)
Age (years)	54.04 ± 11.62
HD vintage (years)	14.1 ± 9.7
Ethnicity	
Malay	55 (49.1)
Chinese	32 (28.6)
Indian	24 (21.4)
Others	1 (0.9)
Primary Cause of ESKD	
Diabetes Mellitus	25 (22.3)
Hypertension	32 (28.6)
Chronic Glomerulonephritis	15 (13.4)
Systemic Lupus Erythematous	2 (1.8)
Renal calculi/ Reflux nephropathy	3 (2.7)
Drug induced	1 (0.9)
Small kidneys	1 (0.9)
Unknown	29 (25.9)
Others	4 (3.5)
No. of patients who had RNA test prior to recruitment	22 (19.6)

We analysed the HCV Ag test comparing to HCV RNA test as gold standard test, as shown in Table 2. Among 86 HCV RNA positive subjects, 78 were HCV Ag positive, giving a sensitivity of 90.7% (95% confidence interval 82.5 to 95.9%) and a specificity of 100% (95% CI 86.8 to 100%). The positive predictive value and negative predictive value were 100 and 76.5% (95% CI 62.7 to 86.3%) respectively, with accuracy of 92.9% (95% CI 86.4 to 96.9%). The Spearman Correlation coefficient was 0.833 (*p* < 0.001).

**Table 2:** Comparison between HCV Ag & HCV RNA test

HCV Ag test result	HCV RNA test result		Total
	Positive	Negative	
Reactive	78	0	78
Non-reactive	8	26	34
Total	86	26	112

We also analyzed the HCV Ag result according to different HCV RNA concentrations as shown in Table 3. There was total of 112 samples where 78 were HCV Ag reactive and 34 non-reactive. All HCV RNA level < 21 IU/mL were HCV Ag negative, whereas among 81 HCV RNA level > 3000 IU/mL, 95.1% of those were HCV Ag positive. Of the 4 HCV Ag negative samples, they have HCV RNA level of 5400, 11,970, 13,770, and 24,840 IU/mL respectively.

**Table 3:** Comparison of HCV Ag among different HCV RNA concentrations

HCV RNA Level (IU/mL)	HCV Ag Test		Total
	Reactive	Non-reactive	
< 21	0	26	26
22–2999	1	4	5
> 3000	77	4	81
Total	78	34	112

Within the HCV RNA level of 22–2999 IU/mL, only one out of five was HCV Ag positive. The positive HCV Ag samples had HCV RNA level of 500 IU/mL whereas the four HCV Ag negative samples had HCV RNA level was 180, 540, 630, 1980 IU/mL respectively.

HCV RNA and HCV Ag both showed a significant correlation at level above 3000 IU/mL. The Spearman test showed a higher correlation with a correlation coefficient of 0.897 (*p* < 0.001).

Among the 8 false negative HCV Ag result, all patients had normal liver function test.

## Discussion

This study indicates a good correlation between the index test, HCV Ag with the gold standard HCV RNA among haemodialysis population. Among 112 anti-HCV seropositive samples, HCV Ag was positive in 90.7% of samples tested positive for HCV RNA. HCV Ag had 8 (9.3%) false negative cases and 0 (0.00%) false positive cases at the cutoff point of 3 fmol/L. This gives a specificity of 100% and a sensitivity of 90.7%. A lower detection rate compared to HCV RNA is consistent with previous studies [7, 9, 10, 17, 19]. Both HCV Ag and HCR RNA tests showed a significant correlation, with Spearmen test coefficient of 0.833 (*p* < 0.001). Our result was reported qualitatively as reactive if HCV Ag value exceeds 3 fmol/L and the correlation result was similar compared to previous studies that reported HCV Ag quantitatively [6, 14].

Furthermore, HCV Ag showed a higher sensitivity rate of 95.1% at greater HCV RNA level (> 3000 IU/mL). The correlation at HCV RNA level above 3000 IU/mL was higher, with Spearman test coefficient of 0.897 (*p* < 0.001). This was similar to the other studies [6, 10], where the sensitivity of HCV Ag increased in higher HCV RNA concentration levels. Among the 4 false negative cases with HCV RNA level > 3000, the HCV RNA levels ranged between 5400 to 24,840 IU/mL, which might be attributed to the types of genotype, as seen in other studies [20, 21].

According to *Trevizoli* et al., HCV RNA levels tend to be lower in patients on long term HD compared to patients with normal renal function [22], which has led to the concern on the suitability of HCV Ag in diagnosing active HCV infection among HD population as the sensitivity of HCV Ag depends on the RNA level [23]. In our study, the median HCV RNA level was 175,455 IU/mL, with only five cases had HCV RNA below 3000 IU/mL. Considering the relatively high viral load among most patients, HCV Ag could potentially be used as another method to diagnose active HCV infection in HD population.

Of those with HCV RNA level less than 3000 IU/mL, HCV Ag was positive in only one sample. HCV RNA for the five samples ranged from 180 to 1980 IU/mL, which was much lower compared to 3000 IU/mL where HCV Ag correlated best with HCV RNA. The four false negative results with HCV RNA level lower than 3000 IU/mL, and the four false negative results with HCV RNA level higher than 3000 IU/mL, have normal liver function test. Hence, all eight results would require a confirmatory molecular RNA testing to resolve this result. In view of the 100% specificity, only HCV Ag negative results require further evaluation by HCV RNA. This would confirm the active infections and determine those who are potential candidates for DAA treatment [19, 24–26].

As far as we can tell, our study is the first study utilizing ARCHITECT HCV Ag test involving Asian populations with the largest HCV seropositive sample size (112) among HD population. It showed that HCV Ag correlates well with HCV RNA in Asian population. However, our study has several limitations. Firstly, despite the largest HCV seropositive study among HD population, the sample size is still rather small. The data on HCV genotype is lacking and may account for the false negative HCV Ag result, particularly amongst those with HCV RNA of higher than 3000 IU/mL [20, 21].

HD population poses a higher risk of HCV infection as compared to the general population. In Malaysia, the prevalence of HCV seropositive among HD population is 2%, in comparison to 0.98% in the general population [27, 28]. From our study, the prevalence of active HCV viremia among HD population is 76.8% among those who are anti-HCV positive. This finding is similar to *Jasuja et.al* who reported 72.7% of active HCV viremia among HCV seropositive HD population [29].

High prevalence of active HCV viremia among HD population is associated with greater risk of HCV transmission. In this study, we demonstrated that only 19.6% (22/112) of HCV seropositive patients have had previous HCV RNA testing. Lack of HCV confirmation poses a great challenge in managing HCV infection and achieving micro-elimination in the HD population. As a LMICs, high cost of HCV confirmatory testing, as well as limited use of HCV DAA therapy in chronic kidney disease patients, was believed to be the major reasons for poor HCV treatment uptake amongst patients with advanced CKD [6, 23].

Based on our study, HCV Ag provides a suitable alternative to HCV RNA due to its lower cost and fast analytical process (within 40 mins) and therefore, short turnaround time [15, 19, 23]. We suggest initial screening by Anti-HCV Ab followed by HCV Ag test for seropositive HD cohorts. For seropositive HD patient with negative HCV Ag, we recommend to follow-up with HCV RNA test as it has negative predictive value of 76.5%. This algorithm, as proposed by *Julicher* et al. [16], and *Wasitthankasem* et al. [17], could potentially be cost-effective as not all seropositive HD patient is subjected to expensive RNA test. This would be a thought-provoking domain for a future study.

## Conclusion

HCV Ag is sensitive and specific to diagnose chronic active HCV infection among haemodialysis patients. Hence, HCV Ag test can be included in the current diagnostic guideline along with anti-HCV and HCV RNA to diagnose chronic active HCV infection among haemodialysis population. This would allow clinician to identify patients eligible for HCV treatment effectively and efficiently.

## Supplementary Information

**Additional file 1.** Testing algorithm; Algorithm of different HCV Ag level and requirement required to repeat testing.

## Data Availability

The datasets used and/or analysed during the current study are available from the corresponding author on reasonable request.

## Abbreviation

CMIA: Chemiluminescent microparticle immunoassay
DAA: Direct-acting Antiviral
HCV: Hepatitis C Virus
HCV Ab: Anti-HCV Antibodies
HCV Ag: HCV Core Antigen
HCV RNA: HCV Ribonucleic acid
HD: Haemodialysis
LMICs: Low- and middle-income countries
NPV: Negative Predictive Value
PCR: Polymerase Chain Reaction
PPV: Positive Predictive Value
WHO: World Health Organization
