# Supplementary material for: Hepatitis C core antigen testing to diagnose active hepatitis C infection among haemodialysis patients
Source: BMC Nephrol. 2020 Nov 13;21:480. doi: 10.1186/s12882-020-02154-4 (PMC7666439; doi:10.1186/s12882-020-02154-4)
Supplement: Supplementary file 1 — Additional file 1. Testing algorithm; Algorithm of different HCV Ag level and requirement required to repeat testing. [file 12882_2020_2154_MOESM1_ESM.docx]

Negative

Positive

>3 fmol/L

<3 fmol/L

<3 fmol/L

3 to 10 fmol/L

Check HCV RNA

Check HCV RNA

Retest HCV Ag

Test for HCV Core Antigen
(HCV Ag)

Hemodialysis patients with positive anti-HCV antibody
